# The Physiological Effect of n-3 Polyunsaturated Fatty Acids (n-3 PUFAs) Intake and Exercise on Hemorheology, Microvascular Function, and Physical Performance in Health and Cardiovascular Diseases; Is There an Interaction of Exercise and Dietary n-3 PUFA Intake?

**DOI:** 10.3389/fphys.2019.01129

**Published:** 2019-08-30

**Authors:** Marko Stupin, Aleksandar Kibel, Ana Stupin, Kristina Selthofer-Relatić, Anita Matić, Martina Mihalj, Zrinka Mihaljević, Ivana Jukić, Ines Drenjančević

**Affiliations:** ^1^Institute and Department of Physiology and Immunology, Faculty of Medicine Osijek, Josip Juraj Strossmayer University of Osijek, Osijek, Croatia; ^2^Croatian National Scientific Center of Excellence for Personalized Health Care, Josip Juraj Strossmayer University of Osijek, Osijek, Croatia; ^3^Department of Cardiovascular Diseases, Osijek University Hospital, Osijek, Croatia; ^4^Department of Pathophysiology, Physiology and Immunology, Faculty of Dental Medicine and Health Osijek, Josip Juraj Strossmayer University of Osijek, Osijek, Croatia; ^5^Department of Internal Medicine, Faculty of Medicine Osijek, Josip Juraj Strossmayer University of Osijek, Osijek, Croatia; ^6^Department of Dermatology, Osijek University Hospital, Osijek, Croatia

**Keywords:** n-3 PUFAs, exercise, cardiovascular, endothelium, inflammation, hemorheology, muscle, microcirculation

## Abstract

Physical activity has a beneficial effect on systemic hemodynamics, physical strength, and cardiac function in cardiovascular (CV) patients. Potential beneficial effects of dietary intake of n-3 polyunsaturated fatty acids (n-3 PUFAs), such as α-linolenic acid, eicosapentaenoic acid, and docosahexaenoic acid on hemorheology, vascular function, inflammation and potential to improve physical performance as well as other CV parameters are currently investigated. Recent meta-analysis suggests no effect of n-3 PUFA supplementation on CV function and outcomes of CV diseases. On the other hand, some studies support beneficial effects of n-3 PUFAs dietary intake on CV and muscular system, as well as on immune responses in healthy and in CV patients. Furthermore, the interaction of exercise and dietary n-3 PUFA intake is understudied. Supplementation of n-3 PUFAs has been shown to have antithrombotic effects (by decreasing blood viscosity, decreasing coagulation factor and PAI-1 levels and platelet aggregation/reactivity, enhancing fibrinolysis, but without effects on erythrocyte deformability). They decrease inflammation by decreasing IL-6, MCP-1, TNFα and hsCRP levels, expression of endothelial cell adhesion molecules and significantly affect blood composition of fatty acids. Treatment with n-3 PUFAs enhances brachial artery blood flow and conductance during exercise and enhances microvascular post-occlusive hyperemic response in healthy humans, however, the effects are unknown in cardiovascular patients. Supplementation of n-3 PUFAs may improve anaerobic endurance and may modulate oxygen consumption during intense exercise, may increase metabolic capacity, enhance endurance capacity delaying the onset of fatigue, and improving muscle hypertrophy and neuromuscular function in humans and animal models. In addition, n-3 PUFAs have anti-inflammatory and anti-nociceptive effects and may attenuate delayed-onset muscle soreness and muscle stiffness, and preserve joint mobility. On the other hand, effects of n-3 PUFAs were variably observed in men and women and they vary depending on dietary protocol, type of supplementation and type of sports activity undertaken, both in healthy and cardiovascular patients. In this review we will discuss the physiological effects of n-3 PUFA intake and exercise on hemorheology, microvascular function, immunomodulation and inflammation and physical performance in healthy persons and in cardiovascular diseases; elucidating if there is an interaction of exercise and diet.

## Introduction

It is well-accepted that physical activity has a beneficial effect on systemic hemodynamics, physical strength and cardiac function in cardiovascular (CV) patients (Joyner and Green, 2009; Smith et al., 2011c; Piepoli et al., 2016). The strength of such evidence is evident from the fact that regular physical activity and exercise are accepted as essential components for reducing the severity of CV risk factors and are incorporated in guidelines for primary and secondary cardiovascular disease (CVD) prevention by both European Society of Cardiology and American Heart Association (Smith et al., 2011c; Piepoli et al., 2016). Even though the effects of regular exercise are more difficult to apprehend in secondary than in primary CVDs prevention (studies performed as part of rehabilitation programs), the current position is that mild-to-moderate regular physical activity can be recommended to CVD patients with no exception (Smith et al., 2011c; Piepoli et al., 2016). In the last few decades, potential beneficial effects of dietary or supplementary daily intake of n-3 polyunsaturated fatty acids (n-3 PUFAs), such as α-linolenic fatty acid (ALA), eicosapentaenoic acid (EPA), and docosahexaenoic acid (DHA), on CV function and outcomes of CVDs (Auger et al., 2016), particularly hemorheology, vascular function, immunomodulation, inflammation and potential to improve muscular strength, have been investigated in different study populations (e.g., healthy, sedentary, athletes, CV patients). On the other hand, recent Cochrane review assessed the effects of intake of fish- and plant-based n-3 PUFA supplements, or in some cases enriched food or dietary advice (n-3 PUFAs doses ranged from 0.5 to >5 g/day) on CV events, adiposity, lipids, and all-cause mortality (Abdelhamid et al., 2018). That review evaluated 79 randomized controlled trials (RCTs) (with subsequent excluding of 25 RCTs), which included approx. 100,000 adults at different CV risks, mainly from high-income countries. Authors concluded that meta-analysis and sensitivity analyses in their review suggested little or no effect of increased DHA/EPA intake on all-cause mortality (high-quality evidence), CV mortality, CV events (high-quality evidence), coronary heart disease (CHD) mortality, stroke or arrhythmia. They may reduce CHD events; however, according to sensitivity analyses – this is small effect. All evidence was of moderate GRADE quality, except ones noted as high quality evidence. However, increased ALA may slightly reduce risk of CV events (low-quality evidence) and probably reduces risk of arrhythmia, while effects on stroke are unclear. Taken together, it seems that n-3 PUFAs and exercise *per se* may affect hemorheology and thrombotic as well as inflammatory status of the body and have protective effect, for example in atherosclerosis, which was not assessed in the aforementioned Cochrane review. Interaction of n-3 PUFA intake and life style (such as physical activity) was not included in the scope of the review by Abdelhamid et al. (2018).

This, in fact, brings some controversies in the field, since the consumption of such remedies is rather high in United States (Hopp and Shurtleff, 2018), while in EU countries the recommendations for daily n-3 PUFA intake are not always met in all population subgroups (Sioen et al., 2017). According to the 2012 National Health Interview Survey in the United States; 7.8% of adults (18.8 million) and 1.1% of children age 4 to 17 (664,000) had taken a fish oil supplement in the previous 30 days (Hopp and Shurtleff, 2018).

The aim of the present review was to summarize, in an orderly manner, current knowledge on the effect of exercise and dietary intake of n-3 PUFAs (in food stuff or in the form of supplements) on vascular function and physical performance in healthy persons and in CV patients, with particular attention paid to hemorheology and coagulability, inflammation and vascular and muscular function.

**FIGURE 1:**
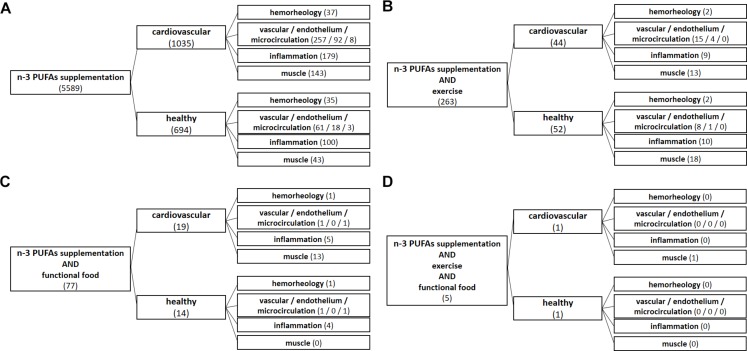
Algorithms of literature search. From literature search it is evident that a respective number of studies investigated the effect of n-3 PUFA supplementation on hemorheology, vascular/endothelial function/microcirculation, inflammation, and skeletomuscular system in both cardiovascular patients and healthy population **(A)**. However, a significantly smaller number of studies dealt with the effect of n-3 PUFA supplementation in the form of functional food **(C)**, or the potential combined interaction effect of n-3 PUFAs and regular exercise on the mentioned parameters **(B)**. Importantly, there is no available data (a total of 5 search results) on the combined effect of n-3 PUFA supplementation in the form of functional foods and regular aerobic exercise on hemorheology, vascular/endothelial function/microcirculation and inflammation in both healthy population and CV patients **(D)**.

To obtain a comprehensive review of current data, original research studies, narrative reviews and systematic reviews and meta-analyses were collected and analyzed. A search of PubMed database was performed by using the following search terms: omega-3 supplementation, n-3 PUFA supplementation, exercise, functional food, cardiovascular, healthy, vascular, endothelium, microcirculation, inflammation, hemorheology, and muscle (Figure 1). Only the English language literature pertaining to both humans and experimental animals with no time restriction were reviewed. Literature search algorithms and obtained results (number of articles) are described in Figure 1. From the literature search it is evident that a respective number of studies investigated the effect of n-3 PUFA supplementation on hemorheology, vascular/endothelial function/microcirculation, inflammation, and skeletomuscular system in both CV patients and healthy population (panel A). However, a significantly smaller number of studies dealt with the effect of n-3 PUFA supplementation in the form of functional food (panel C), or the potential combination intervention effect of n-3 PUFAs and regular exercise on mentioned parameters (panel B). Importantly, there is no available data (total of 5 research results) on the combined effect of n-3 PUFA supplementation in the form of functional foods and regular aerobic exercise on hemorheology, vascular/endothelial function/microcirculation and inflammation in both healthy population and CV patients (panel D).

## Metabolism of Polyunsaturated Fatty Acids

**FIGURE 2:**
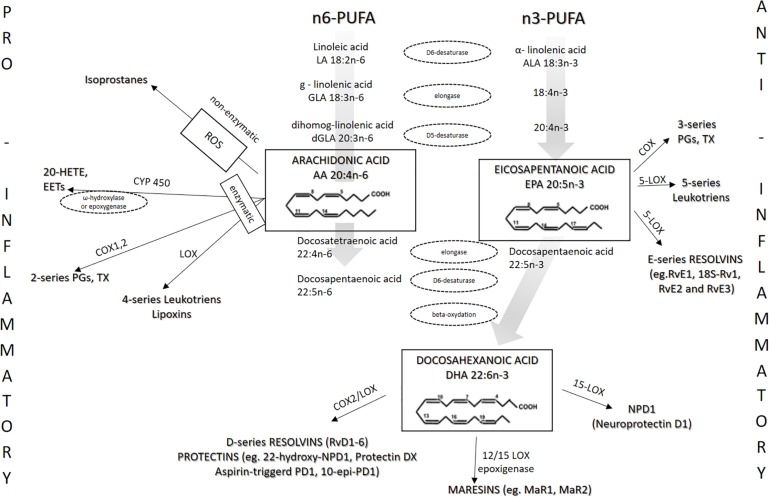
Metabolism of n-3 and n-6 PUFAs and the most important eicosanoids. The present figure summarizes the metabolism of n-3 PUFAs and n-6 PUFAs by cyclooxygenases (COX), lipoxygenase (LOX), and cytochrome P450.

EPA and DHA are present in phospholipids of the cell membrane, contributing to the n-3/n-6 ratio, and share the same enzymes with arachidonic acid (AA) in the eicosanoid-producing process (Wang et al., 2017; Gammone et al., 2018; Philipsen et al., 2018). EPA and DHA are metabolized into numerous eicosanoids and docosanoids, respectively, by cyclooxygenases (COX), lipoxygenase (LOX), and cytochrome P450 (Figure 2). Because n-3 PUFAs can compete for the same metabolic pathways against n-6 PUFAs, n-3 PUFAs supplementation may also affect the metabolism of AA, thereby shifting the profile of metabolites derived from AA (Figure 2) (Drenjančević et al., 2017).

The AA-derived prostaglandins and leukotrienes are potent pro-inflammatory mediators (Samuelsson et al., 1987), whereas the AA-derived metabolites lipoxins are potent anti-inflammatory and pro-resolving molecules (Serhan, 1994; Wallace and Fiorucci, 2003; McMahon and Godson, 2004). On the other hand, anti-inflammatory lipid mediators or specialized pro-resolving mediators (SPM), such as lipoxins, resolvins, protectins, and maresins, are endogenous n-3 PUFA-derived oxygenated metabolites of EPA, docosapentaenoic acid (DPA) and especially DHA. They are released upon specific stimuli and mediate cell signaling and cell-to-cell interactions. Mostly, they are responsible for regulating inflammation and specific immune responses (Calder, 2017). Addition of n-3 PUFA, particularly EPA and DHA supplements into the diet leads to their increased incorporation into cell membrane phospholipids at the expense of n-6 PUFAs, such as AA. EPA as a 20-carbon chain fatty acid competes directly with AA as a substrate for COX and LOX and leads to production of anti-inflammatory (less inflammatory) prostaglandins and leukotrienes (i.e., LTB3 and PGE3), while DHA, together with EPA, has been associated with production of counter-regulatory lipid mediators such as resolvins, protectins, and maresins (Calder, 2017; Molfino et al., 2017). Maresin 1 is biosynthesized via LOX by DHA to generate 14S-hydroperoxydocosa-4Z,7Z,10Z,12E,16Z,19Z hexaenoic acid, which undergoes further conversion via epoxidation in macrophages and is subsequently converted to 7R,14S-dihydroxydocosa-4Z,8Z,10E,12Z,16Z,19Z-hexaenoic acid, known as maresin 1 (MaR 1) (Serhan et al., 2012). Sun et al. (2017) recently showed that MaR 1 exhibited its protective effects, at least in part, via the Nrf-2-mediated heme oxygenase-1 (HO-1) signaling pathway. MaR 1 can directly modulate the inflammatory responses by affecting the function of already activated and clonally expanded Th1 and Th17 cells, and additionally influence immune responses by acting on their transcription factor-induced activation programs to prevent their generation from naïve CD4 + T cells. Furthermore, MaR 1 can enhance the differentiation of CD4 + T cells into Treg cells (Chiurchiù et al., 2016). They also showed that the protective effect of MaR 1 relied on its downstream antioxidative stress capabilities and and capability to maintain the balance between oxidative and antioxidative stress. MaR 1 significantly reduced reactive oxygen species, malondialdehyde, and 15-F2t-isoprostane generation and restored the activity of antioxidative enzymes (superoxide dismutase, glutathione peroxidase, catalase) (Sun et al., 2017).

Beside reduced production of potent pro-inflammatory AA-derived eicosanoids such as PGE2, anti-inflammatory action of EPA-derived eicosanoids is reflected in the fact that they have reduced affinity for eicosanoid receptors. For example, Wada et al. (2007) described that PGE_3_ has 50 to 80% lower potency compared with PGE_2_ toward the EP_1_, EP_2_, EP_3_, and EP_4_ receptors. In addition, increased EPA and DHA cellular content results in decreased expression of COX-2 enzyme (Jeromson et al., 2015; Gammone et al., 2018). Figure 2 shows the metabolism of n-3 and n-6 PUFAs and the most important eicosanoids involved in the processes of inflammation, vascular reactivity, thrombosis, and similar.

## Effects of n-3 Pufas and Exercise on Hemorheology and Coagulability

### Supplementation or Dietary Intake of n-3 PUFAs Affects Hemorheology and Coagulability

Dietary n-3 PUFAs are associated with a hypocoagulable profile. In the Atherosclerosis Risk in Communities (ARIC) Study, four population-based samples amounting to over 15,000 participants, blacks and whites, have been studied (Shahar et al., 1993). This study assessed if dietary fatty acid ingestion leads to changes in blood concentrations of relevant coagulation factors. The investigators analyzed concentrations of fibrinogen, factor VII, factor (vWF), protein C, and antithrombin III. A food frequency questionnaire was used to document dietary intake. Multiple linear regression was used to assess cross-sectional associations, with adjustments for age, race, gender, smoking and alcohol use, diabetes, body mass index, and field center. Dietary n-3 PUFAs have been shown to be negatively associated with concentrations of fibrinogen, factor VIII, and (vWF) (in black and white individuals) and are positively associated with protein C (in white subjects only). The ingestion of fish, which represented the predominant source of dietary n-3 PUFAs, was similarly related to the hemostatic profile. Namely, fish ingestion that was 1 serving higher per day showed an association with these predicted differences (95% confidence interval): factor VIII, −3.3% (−5.4, −1.3); fibrinogen, −2.9 mg/dL (−6.3, 0.5); vWF, −2.7% (−5.2, −0.1) (in black and white subjects); and protein C, +0.07 μg/mL (0.03, 0.11) (white subjects only). Other analyzed nutritional items were variably correlated with the levels of hemostatic factors. The results of the ARIC study with its associations that are population-based, albeit cross-sectional, imply that elevations in dietary intake of n-3 PUFAs from fish might lead to alterations of blood concentrations of several important coagulation factors. These associations are tied to the hypocoagulable profile (Shahar et al., 1993). Phang et al. (2014) performed a double-blinded, placebo-controlled randomized trial in 94 healthy adults, assessing platelet coagulation parameters such as factors I, II, V, VII, VIII, IX, X, vWF and endogenous thrombin potential, as well as platelet aggregation. They found that supplementation with EPA in healthy men and DHA in women leads to reduction in platelet aggregation. Similarly, plasma concentrations of factor II, factor V and vWF were reduced primarily in men with EPA supplementation, while reduction in platelet aggregation mediated by DHA in women was not associated with substantial changes in the assessed parameters of coagulation (Phang et al., 2014). This brings to light an interesting sex-specific difference in the antithrombotic effects of n-3 PUFAs.

The addition of n-3 PUFAs to simvastatin treatment in patients with combined hyperlipidemia lead to a reduction of the fraction of the free tissue factor pathway inhibitor in the fasting state. This n-3 PUFA addition also inhibited factor VII activation during post-prandial lipemia, as found by Nordøy et al. (2000) on a sample of 41 patients. A double-blind, cross-over study using olive oil as placebo, assessed the influence of a daily dosage of 6 g of fish oil on CV risk markers of 20 healthy young volunteers (10 men and 10 women). This study implicated a reduction in factor X (as a result of fish oil ingestion in women, compared to placebo) (Oosthuizen et al., 1994), but there are also inconsistencies in the findings, since some studies did not detect clear changes in the levels of coagulation factors (Vanschoonbeek et al., 2004) or platelet function with n-3 PUFA ingestion (Lee et al., 2006; Poreba et al., 2017).

A decrease in thromboxane A2 synthesis, which is an important platelet aggregation facilitator, contributes to the hypocoagulable state induced by n-3 PUFAs (Véricel et al., 2015; Lands, 2016; Jeansen et al., 2018). EPA and DHA are also antagonists of human platelet thromboxane A2 and prostaglandin H2 receptors (Véricel et al., 2015; Lands, 2016; Jeansen et al., 2018). In addition, n-3 PUFAs lead to a decrease in plasma viscosity in patients with hypolipoproteinemia by altering the protein pattern of the plasma (Ernst, 1989).

There is evidence that the n-3 PUFA ALA, which is converted to EPA and DHA, impairs formation of arterial thrombus, expression of tissue factor, and platelet activation in male C57Bl/6 mice who were fed a high-ALA diet for 2 weeks (Holy et al., 2010). Furthermore, there is also clinical evidence regarding the influence of n-3 PUFAs on clot properties. For instance, a study by Gajos et al. (2011) assessed the effects of n-3 PUFAs on top of dual antiplatelet therapy in stable coronary artery disease patients undergoing percutaneous coronary intervention. The addition of n-3 PUFAs (supplementation 1 g/day for 1 month) to standard therapy (30 treated subjects and 20 patients on placebo) lead to a decrease in thrombin formation and oxidative stress and favorably altered fibrin clot properties in the treated patients (Gajos et al., 2011).

**FIGURE 3:**
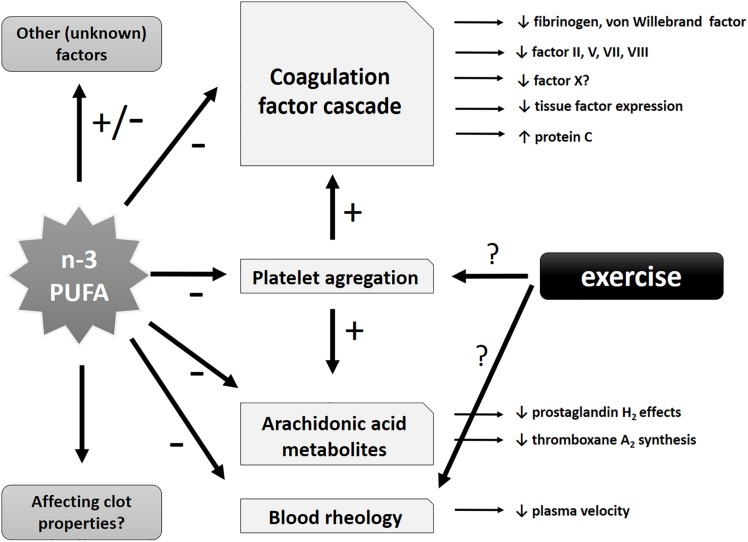
Schematic representation of potential antithrombotic effects of n-3 PUFAs and exercise. Potential effects of n-3 PUFAs may include influence on specific coagulation factors of the coagulation cascade (with antithrombotic tendency), effects on arachidonic acid metabolites, blood rheology and (directly and indirectly) platelet aggregation (leading to a decrease in the latter). Other unknown or insufficiently investigated effects might also be involved.

Regarding the safety of n-3 PUFA consumption, when its antithrombotic effects are taken into consideration, it has been analyzed in various patient populations in clinical settings. An assessment of 8 clinical intervention studies (with over 600 treated individuals) that used nutritional supplements with fish oil as a source of n-3 PUFAs did not find any increased bleeding risk as a result of n-3 PUFA intake. Additionally, no significant changes in crucial parameters of coagulation (partial thromboplastin time and prothrombin time) were observed. Therefore, the authors concluded that n-3 PUFA intake seems to be safe even at a short-term dosage of up to 10 g/day of EPA + DHA or consumed for up to 52 weeks at higher than 1.5 g/day, in selected vulnerable and sensitive patient populations including individuals with gastrointestinal malignancies or intensive care patients (Jeansen et al., 2018). The potential protective antithrombotic effects of PUFAs are summarized in Figure 3.

### Effects of Exercise on Hemorheology and Coagulability in CV Patients

Regarding the influence of exercise on platelet function in patients with CV disease, it is not entirely clear in which way exercise may influence platelets. In their systematic review, Hvas and Neergaard-Petersen analyzed a total of 18 studies from various databases (including PubMed, Embase, Scopus, and Cochrane Library). Of the 18 studies, 7 were with coronary artery disease patients, 5 with angina pectoris patients, 5 with hypertensive patients, and 2 with subjects who have peripheral artery disease (Hvas and Neergaard-Petersen, 2018). They found that conflicting results were reported, with certain studies reporting increased platelet aggregation and/or platelet activation, some studies found no difference, while several reported a reduction in platelet aggregation after exercise (compared with controls). Therefore, more conclusive research is needed to finally elucidate potential effects of exercise on platelet function.

Exercise *per se* has been related to beneficial changes of rheological characteristics of the blood. In a meta-analysis of available studies which investigate the effects of exercise on rheological characteristics of blood, Romain et al. (2011) concluded that regular exercise decreases hematocrit and red blood cell aggregation. However, the main criticism of Romain’s analysis was the small number and a large heterogeneity of the available studies (methodology, study design, etc.) which hampered final conclusions (Romain et al., 2011), resulting in uncertainty of interpretation of data and necessity of future randomized controlled clinical studies for final conclusions.

### Combined Interaction Effect of n-3 PUFA Consumption and Exercise on Hemorheology and Antithrombic Status of Blood in CV Patients

The search for combined interaction effect of n-3 PUFA consumption and exercise on hemorheology and coagulability of blood in CV patients rendered scarce results. It is reported that in previously sedentary, obese people (*N* = 22; 12 women, 10 men, BMI 26.6 ± 0.7 kg/m^2^) 4 weeks of dietary and exercise protocols, exercise training [brisk walking and (or) jogging at 60% VO_2_ maximum for 45 min/day, 5 days/week] has no interference or additive effects with 4 g/day of n-3 PUFA supplementation in attenuating post-prandial lipemia, but combined treatments may be additive in raising high-density lipoprotein (LDL) cholesterol (Thomas et al., 2007), which may help maintain antithrombotic and anti-atherosclerotic status of blood. Obviously, well-planned, controlled trial of combined dietary and exercise protocols focused on blood hemorheology could provide new evidence on interactions of interest.

## The Effect of n-3 Pufas and Exercise on Vascular Function in Healthy Subjects and in Cardiovascular Patients

**FIGURE 4:**
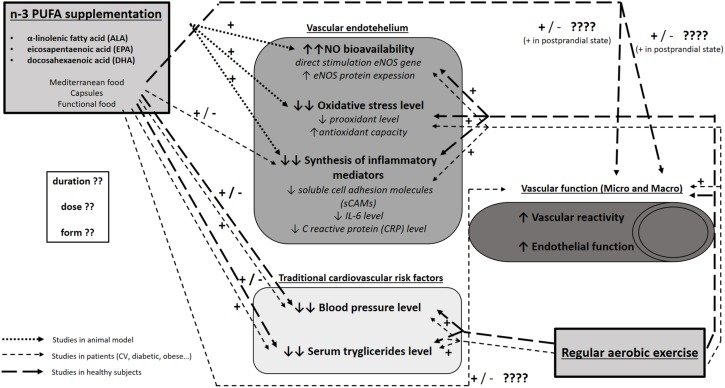
Functional pathways and molecular mechanisms mediating interaction between n-3 PUFAs and exercise on the one side and endothelial function on the other. The present figure summarizes the results of studies conducted on experimental animals, healthy subjects, and patients (CV, diabetic, obese) on the effect of n-3 PUFA supplementation or regular exercise on endothelial function (NO bioavailability, oxidative stress level and inflammation), vascular function (functional vascular experiments in both macro- and micro-circulation), and traditional cardiovascular risk factors (e.g., blood pressure, serum triglyceride level). It is evident that studies on the effect of regular exercise on the abovementioned parameters provided more uniform results and yielded clear conclusions on the beneficial effect of regular exercise on CV health. On the other hand, studies on the effect on n-3 PUFAs on vascular and endothelial function, especially studies in patients and healthy subjects, provided very divergent and inconclusive results, evidently due to the heterogeneity in experimental design with the emphasis on different form, dose and duration of n-3 PUFA supplementation used in these studies. There is a paucity of data on the combined effect of n-3 PUFAs and regular exercise on CV function in general.

Since it is widely accepted that n-3 PUFA consumption reduces CV risk (Auger et al., 2016), numerous studies set their focus on investigating whether n-3 PUFA intake may prevent or delay the initial steps in the pathogenesis of various CV diseases, e.g., changes in endothelium and vascular function, in both healthy and diseased population. In particular, n-3 PUFAs may improve endothelial function by increasing the bioavailability of endothelial nitric oxide (NO) (through increasing its production by stimulating endothelial NO synthase gene and protein expression) (Lo et al., 2004; Gortan et al., 2013; Zhang et al., 2013) or by reducing the level of oxidative stress (through attenuating reactive oxygen species, which indirectly increases NO bioavailability) (Gortan et al., 2013; Zhang et al., 2013; Drenjančević et al., 2017). Additionally, vascular function could be improved by reducing inflammation (by affecting mediators of local endothelial and/or systemic inflammation) (Wang et al., 2011). All of these interactions are summarized in Figure 4.

While there is paucity of data on the effect of n-3 PUFAs on microvascular function in both healthy and diseased population (Pe et al., 2008; Stupin A. et al., 2018), there is large inconsistency of functional vascular studies which evaluated the effects of n-3 PUFAs on macrovascular function, mainly assessed by flow-mediated dilation (FMD) of brachial artery as a gold standard method, in both healthy individuals and CV patients (Egert et al., 2014; Merino et al., 2014; Tousoulis et al., 2014; Yagi et al., 2015).

### Beneficial Effect of n-3 PUFAs on Vascular Function in CV Patients

Studies in CV patients (e.g., coronary artery disease, metabolic syndrome, chronic heart failure) or individuals with increased CV risk (e.g., obesity, diabetes mellitus type 2, hyperlipidemia, cigarette smoking) strongly suggest that n-3 PUFA supplementation improves vascular and endothelial function (e.g., brachial artery FMD, venous occlusion strain-gauge plethysmography, microvascular endothelial cells) (Morgan et al., 2006; Egert et al., 2014; Yagi et al., 2015). For example, oral intake of 2 g of n-3 PUFAs per day for 12 weeks significantly improved FMD of brachial artery and pulse wave velocity in adults with metabolic syndrome (Tousoulis et al., 2014). A recent cross-over study in overweight individuals reported that a walnut-rich diet (23.1% energy from n-3 PUFAs) compared to an almond-rich diet significantly improved FMD of brachial artery and decreased soluble vascular cell adhesion molecule level (Bhardwaj et al., 2018). Furthermore, increased serum DHA level was a positive contributor to an increased FMD of brachial artery in 160 consecutive Japanese patients with coronary artery disease (Yagi et al., 2015). FMD of brachial artery was improved following intake of 1.8 g/day n-3 PUFA supplementation for 12 weeks in hypertensive patients with hypertriglyceridemia and high CV risk as demonstrated by interventional study (Casanova et al., 2016). Similar results were also reported in overweight dyslipidemic patients and type 2 diabetics (Egert et al., 2014; Tousoulis et al., 2014), along with decrease of soluble thrombodulin, the marker of vascular endothelial damage, in patients with the presence of one or more risk factors for atherosclerosis (Kawauchi et al., 2014). Kondo et al. (2014) reported that a 4-week diet period rich in n-3 PUFAs significantly improved reactive hyperemia measured by strain-gauge plethysmography in post-menopausal women with diabetes mellitus type 2. Furthermore, n-3 PUFA supplementation in patients with chronic heart failure improved endothelium-dependent vasodilation measured by venous occlusion strain-gauge plethysmography (Morgan et al., 2006). Beneficial effect of n-3 PUFAs on microvasculature was confirmed in the model of inflammatory bowel disease on a primary culture of human intestinal microvascular endothelial cells (Ibrahim et al., 2011).

### Beneficial Effect of n-3 PUFAs on Vascular Function in Healthy Subjects

There is inconsistency in the results of studies investigating whether the same beneficial effect of n-3 PUFA consumption is also present in healthy population. Although the results of several studies demonstrated that n-3 PUFA supplementation improved brachial artery FMD in healthy individuals (Walser et al., 2006; Shah et al., 2007; Rizzaa et al., 2009), others failed to observe such effect (Sanders et al., 2011; Skulas-Ray et al., 2011; Singhal et al., 2013). The most evidence for the benefits of n-3 PUFAs to vascular function in healthy individuals was observed in the post-prandial state, in both macrocirculation (using FMD) (Fahs et al., 2010; Miyoshi et al., 2014) and microcirculation (using LDF) (Armah et al., 2008; Pe et al., 2008). Supplementation of fish oil (rich in n-3 PUFAs) for 8 months improved endothelial function in normal healthy subjects (Khan et al., 2003). A recent study by our research group reported that young healthy subjects who consumed n-3 PUFA enriched eggs for 3 weeks (777 mg of n-3 PUFAs/day) had improved skin microvascular reactivity in response to vascular occlusion (Stupin A. et al., 2018). On the contrary, a single Mediterranean meal (rich in n-3 PUFAs; 2.29 g of n-3 PUFAs per meal) did not significantly alter FMD in healthy men, while a high-saturated fatty acid meal induced post-prandial endothelial dysfunction (Lacroix et al., 2016) and only n-3 PUFA supplementation higher than 1.8 g per day improved FMD in healthy adults (Sanders et al., 2011).

The results of two large meta-analyses (including both healthy individuals and CV patients) demonstrated that n-3 PUFA supplementation significantly increases FMD of brachial artery, but also showed that the health status of the study population, as well the dose of n-3 PUFA supplementation may modify the effect of n-3 PUFAs on vascular function (Wang et al., 2012; Xin et al., 2012). Furthermore, when the analysis included only double-blinded, placebo-controlled studies, the supplementation of n-3 PUFAs had no significant effect on vascular endothelial function (Wang et al., 2012). Consequently, the results of the abovementioned studies should still be interpreted cautiously, due to the large heterogeneity of these studies in terms of selection of participants, their age and health status, as well as in terms of the dose and form of n-3 PUFA supplementation used in the trials.

### Beneficial Effects of Exercise on Vascular Function in CV Patients and Healthy Persons

Positive effects of moderate physical activity in preserving general health and preventing CV disease and age-related deterioration have been very well-established (Warburton and Bredin, 2017). Regular exercise significantly contributes to lowering arterial blood pressure or reducing blood lipid concentration (Joyner and Green, 2009). Functional vascular studies on the conductance and resistance arteries in CV patients showed decreased inflammation and endothelial dysfunction of the blood vessels (Hambrecht et al., 2003), while 4 weeks of exercise improved FMD of brachial artery in patients with stable coronary disease (Hambrecht et al., 2003).

Similarly, regular exercise has a beneficial effect on vascular function, even in healthy people. For example, athletes have better FMD of brachial artery compared to healthy sedentary individuals (Clarkson et al., 1999; Kasikcioglu et al., 2005). Several mechanisms may be involved in these positive effects of long-term regular physical activity, such as increased NO bioavailability, increased antioxidative defense in the vascular system and reduced levels of locally or systemically derived mediators of inflammation (Padilla et al., 2011). It has been demonstrated that the repeated increase in blood flow (e.g., vascular shear stress) during physical activity is probably a key mechanism that induces a positive adaptation of vascular function to regular physical activity by stimulating the NO-dependent vasodilatory pathway (Laughlin et al., 2008; Birk et al., 2012). Nevertheless, production of COX-derived vascular metabolites, including increased synthesis of prostacyclin, is involved in endothelial shear stress adaptations. Furthermore, increasing evidence suggests that increased shear stress is a signal that will reduce the level of endothelin 1, soluble cell adhesion molecules and various markers of endothelial activation (Himburg et al., 2007), all together contributing to improved macrovascular function.

Functional vascular studies on microcirculation of the skin showed that regular body activity lasting several weeks to several months resulted in the adaptation of skin microcirculation which is manifested by improved vasodilation-dependent endothelium in both healthy population and CV patients. The main evidence derives from vascular experiments studying the microcirculation response to the endothelium-dependent [acetylcholine (ACh)] and/or endothelium independent [sodium nitroprusside (SNP)] vasodilator. Kvernmo et al. (1998) demonstrated better response in terms of microvascular flow in the forearm skin following ACh administration in professional athletes compared with appropriate controls that had moderate body activity, while there was no difference in SNP response between these two experimental groups, suggesting that endothelial function was affected. Similarly, Stupin M. et al. (2018) reported that professional rowers had significantly better response of forearm skin microcirculation to vascular occlusion and ACh, but not SNP compared to sedentary healthy controls. Furthermore, Wang (2005) investigated endothelium-dependent and endothelial-independent vascular response in microcirculation of the skin in healthy sedentary individuals before and after 8 weeks of exercise. The results of this study have also shown a marked improvement in endothelium-dependent vascular response, but without any change in endothelium-independent response of skin microcirculation. Interestingly, in the further course of the study after 8 weeks without exercise such enhanced endothelium-dependent response was no longer present. In general, skin microvascular endothelial function was not different between adult and young active individuals (Black et al., 2008; Tew et al., 2010). On the other hand, older individuals practicing exercise have better endothelial vascular function in microcirculation of the skin compared to their sedentary peers (Black et al., 2008). Similar effects have been reported in patients with type 2 diabetes (Colberg et al., 2002) and chronic venous disease (Klonizakis et al., 2009).

### Interactive Effects of n-3 PUFAs and Long-Term Exercise on Vascular Function in Humans

Taken together, it is evident that both n-3 PUFA supplementation and regular physical activity improve endothelial function in both macro- and microcirculation of healthy individuals, individuals with increased CV risk and CV patients (Morgan et al., 2006; Joyner and Green, 2009; Egert et al., 2014). However, there is a paucity of data on the combination of these two divergent interventions on vascular function in these populations. The only available study at the moment of preparation of the manuscript is one on an experimental animal model by Barbeau et al. (2017), who reported that combining ALA and endurance exercise resulted in additional reduction of CV disease risk compared to individual interventions in the obese Zucker rat. However, there are many potentially common steps and outcomes that have been demonstrated to be modified by exercise and diet *per se*, as schematized in Figure 4. For example, arterial blood pressure and serum lipid concentration are both affected by n-3 PUFAs and exercise. Similarly, oxidative stress, inflammatory, and biomarkers of vascular function are affected independently by n-3 PUFAs and exercise. On the other hand, the interactive effect of n-3 PUFAs and exercise on functional responses of microcirculation is still unknown. Thus, it is tempting to speculate that these two stimuli which both individually result in improved endothelial function would have additive effect on endothelial function and may be a new challenging avenue of research.

## Immunomodulatory and Anti-Inflammatory Properties of n-3 Pufas and Exercise in Athletes and Cardiovascular Patients; Muscle Soreness, Muscle Stiffness, and Joint Mobility

### Immunomodulatory and Anti-inflammatory Properties of n-3 PUFAs

It is widely accepted that inflammation underlies many chronic non-communicable diseases, such as CV diseases, atherosclerosis, Alzheimer’s disease, and cancer (Coussens and Werb, 2002; Libby, 2002; Weiner and Selkoe, 2002). In general, n-3 PUFAs are considered to have anti-inflammatory effects on various biological systems, including skeletal muscles, respiratory mucosa and CV system, all of which have an important role in exercise physiology (Biltagi et al., 2009; Tecklenburg-Lund et al., 2010; Mori, 2014; Jeromson et al., 2015; Gammone et al., 2018). However, our knowledge of immunomodulatory effects of n-3 PUFAs in humans is scarce (Chen et al., 2018; Dátilo et al., 2018; Phitak et al., 2018). Although much is known about the molecular basis of initiating signals and pro-inflammatory chemical mediators in inflammation (Samuelsson, 2012), it has only recently become interesting to explore endogenous stop signals such as lipid mediators. Most inflammatory reactions are self-limited, and, besides leukocytes withdrawal, the process of resolution includes a switch in synthesis of AA-derived pro-inflammatory mediators to anti-inflammatory pro-resolving n3-PUFA-derived mediators. Previous studies have demonstrated that n-3 PUFAs alleviate inflammation by affecting the production of inflammatory mediators such as eicosanoids, reactive oxygen species and cytokines [i.e., tumor necrosis factor α (TNFα), interleukin 1β and interleukin 6 (IL-6)], responsible for release of other inflammatory factors and acute-phase proteins from the liver (Northoff and Berg, 1991; Calder, 2006; Ambrozova et al., 2010; Jouris et al., 2011).

Increased consumption of EPA and DHA has been linked to decreased serum levels of hsCRP in a heterogeneous set of clinical trials (Reinders et al., 2012; Kubota et al., 2015). For example, one interesting observation is that increased n-3 PUFA intake has increased apolipoprotein A1 and decreased high-sensitivity C reactive protein (hsCRP) concentration and serum concentration of soluble cell adhesion molecules and other pro-inflammatory factors in patients at intermediate-high CV risk, resulting in improved peripheral vasoactivity (Merino et al., 2014). In a cross-sectional study on 992 patients with stable coronary artery disease, a multivariable linear regression model demonstrated that levels of n-3 PUFAs (DHA + EPA) were inversely and independently associated with CRP and IL-6 concentration (Farzaneh-Far et al., 2009). Regarding other inflammatory parameters that were assessed, results are mixed and dependent on the extent of obesity, as beneficial effects were only observed in severely, but not moderately obese individuals (Itariu et al., 2012; Kratz et al., 2013). Furthermore, n-3 PUFAs affect the life and function of lymphocyte subpopulation; administration of n-3 PUFAs decreases lymphocyte proliferation, and alters neutrophil and natural killer (NK) cell function (Varming et al., 1995; Thies et al., 2001a, b).

Transcription factor nuclear factor κB (NF-κB) has a central role in the initiation of an immune response by inducing transcription of genes encoding various cytokines (i.e., IL-1, IL-6, TNFα), chemokines, cell adhesion molecules, anti-apoptotic factors, cell cycle regulators (i.e., cyclin D), and other cell-type specific mediators of inflammation (i.e., inducible NO synthase, COX2) (Afonina et al., 2017). EPA and DHA are natural agonists of transcription factor peroxisome proliferator-activated receptor γ (PPAR-γ), whose activation potently inhibits cytokine-induced NF-κB transcriptional activity in skeletal muscles in a time- and dose-dependent manner, as evidenced by significantly reduced levels of TNFα, IL-8, intercellular adhesion molecule 1 and chemokine (C-X-C motif) ligand 1 (Grygiel-Górniak, 2014).

### Immunomodulatory and Anti-inflammatory Properties of Exercise

Prolonged and exhaustive exercise, but not moderate exercise, leads to a transient increase in frequencies in peripheral blood lymphocytes during the exercise, possibly due to the mobilization of CD4 + T cells, CD8 + T cells, CD19 + B cells, CD16 + NK cells, and CD56 + NK cells from the peripheral lymphoid organs, which is followed by a significant decline in total blood lymphocyte numbers immediately after cessation of exercise. Some of these changes have been attributed to elevated levels of catecholamines during intense training. Furthermore, heavy training loads inhibit NK and B cells activity, and promote Th2 response characterized by Th2 cells and Treg expansion. Levels of pro-inflammatory cytokines and chemokines (i.e., IL-1, IL-6, TNF-α, MIP-1, IL-8) rise during intense training and some remain elevated for hours after the acute intense bout of training. In the humoral immune response, reduced antibody production and secretion of antibodies to the mucosa have been observed (Pedersen, 2000; Shaw et al., 2018). These changes could lead to defective immune responses and make athletes prone to infection.

**FIGURE 5:**
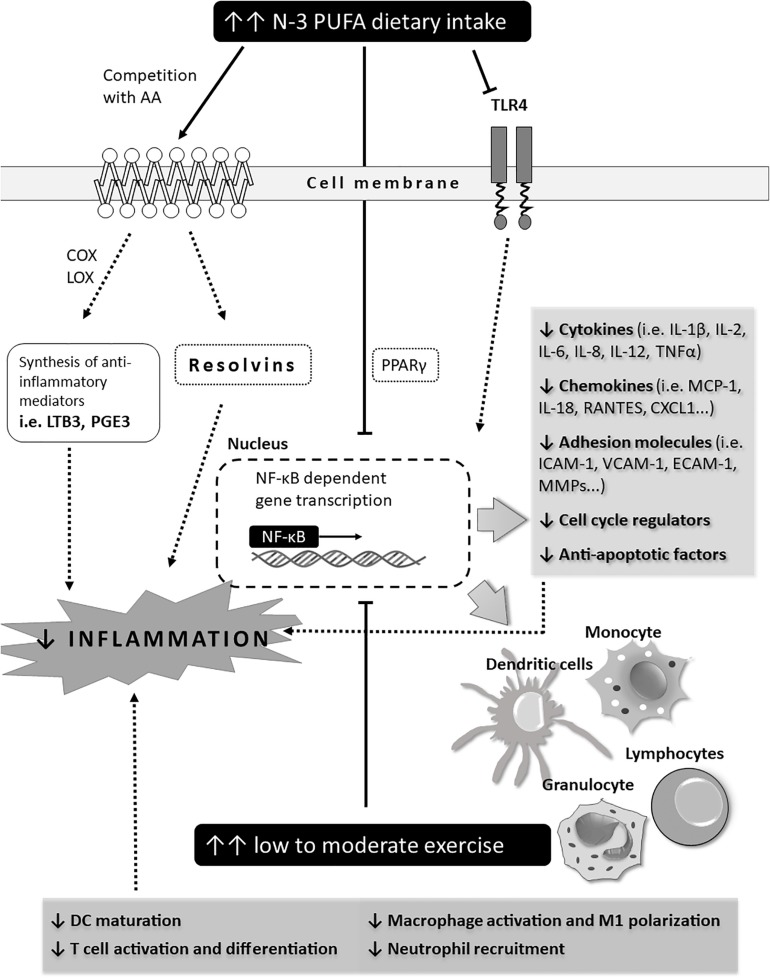
Schematic representation of potential immune mechanisms affected by n-3 PUFAs and exercise in CV patients (outcomes). The effect of exercise and n-3 PUFAs on NF-κB transcriptional activity. Effect of exercise is dependent on the type and intensity level of physical activity. An acute bout of exercise activates myocardial NF-κB and increases toll-like receptor 4 signaling leading to inflammation while moderate exercise reduces NF-κB signaling and activates the SIRT1-AMPK-PGC1α axis, resulting in decreased inflammation and reduced muscle loss.

The effect of exercise on NF-κB transcriptional activity is dependent on the type and intensity of physical activity. An acute bout of exercise activates myocardial NF-κB and increases toll-like receptor 4 signaling leading to inflammation (Balan and Locke, 2011; Cristi-Montero et al., 2012; Vella et al., 2012), while moderate exercise reduces NF-κB signaling and activates the SIRT1-AMPK-PGC1α axis, resulting in decreased inflammation and reduced muscle loss (Liu and Chang, 2018). All of this has been summarized in Figure 5.

### Combined Interaction Effect of n-3 PUFAs and Exercise on Immunomodulation

It is interesting that n-3 PUFAs from fish oil have been found to reduce serum cortisol levels and the production of TNF-α and IL-8 after strenuous exercise. Fish oil supplementation has no effect on lymphocytosis observed during strenuous exercise; however, it prevented the post-exercise decrease in CD8 + T cells (Peres et al., 2018). Four weeks of n-3 PUFA supplementation attenuates the increase in serum inflammatory markers during a period of 1 to 4 days after eccentric exercise (DiLorenzo et al., 2014). DHA supplementation reduces exercise-induced muscle soreness and stiffness, which could be beneficial for improving tolerance to new and/or strenuous exercise programs and achieving better training adaptations (DiLorenzo et al., 2014; Corder et al., 2016). Furthermore, n-3 PUFAs exhibit an antinociceptive effect by binding to the receptors in nervous tissue (Nakamoto et al., 2011). Additional mechanisms of n-3 PUFA-mediated pain control include decreased pro-inflammatory cytokine secretion (Zaloga and Marik, 2001) and/or blockage of cationic channels (Xiao et al., 1995). Other possible mechanisms of decreased nociception include direct stimulation of opioid receptors or alteration of plasma levels of endogenous opioid peptides (Nakamoto et al., 2011). Based on these findings, it seems plausible that n-3 PUFA supplementation could be useful for preventing inflammation and delayed-onset muscle soreness (DOMS) resulting from exhausting exercise (Corder et al., 2016).

In athletes, n-3 PUFAs have potentially beneficial effects on the immune system and inflammation, in terms of alleviation of the observed post-exercise immunosuppression and anti-inflammatory effects that reduce exercise-induced DOMS and muscle stiffness. The consumption of omega 3 fatty acids modulates Th1/Th2 balance and leads to an increase in the plasma levels of ALA, EPA and DHA, and proportionally to the reduction in the levels of AA. Such system leads to a decrease in the production of inflammatory lipid mediators which provide a high-level immune response after exhaustive training. Consumption of fish-oil supplementation at a dose of 1.8 g/day during 6 weeks significantly reduced prostaglandin E2, interferon-γ, and TNFα concentration in elite swimmers (Andrade et al., 2007). Consumption of 1.6 g/day of n-3 PUFAs combined with a single bout of endurance exercise increased the level of IL-2 production and NK cell cytotoxic activity 3 h after the exercise in healthy men, suggesting interactive effects of n-3 PUFAs and exercise on the native immune system (Benquet et al., 1994; Kawabata et al., 2014; Da Boit et al., 2017). Reduced pro-inflammatory production of cytokines, decreased neutrophil function, and NK cell cytotoxicity is established in cases of a single bout of high-intensity or long-duration endurance exercise (Gleeson et al., 2011; Gammone et al., 2018).

Increased oxidative stress induced by exercise is accompanied by a reduced immune response and decreased resistance to infection (Sen, 1995) and makes athletes prone to upper respiratory tract infections. Furthermore, exercise-induced oxidative stress and inflammation, if prolonged, might give rise to systemic inflammation, insulin resistance, and diabetes mellitus type 2 (Bogdanovskaya et al., 2016). At that point, n-3 PUFAs may become important mediators in the prevention of infection (Gray et al., 2012). This is supported by the findings that 6 weeks of consumption of 3 g/day of fish oil (double-blind, randomized, placebo-controlled trial) increased post-exercise production of IL-2 in peripheral blood mononuclear cells and NK cell activity (Gray et al., 2012). In another study, n-3 PUFA dietary supplementation reduced bronchial inflammation by altering immune cell composition in the sputum and reduced pro-inflammatory eicosanoids and IL-1 and TNFα production (Schubert et al., 2009). In addition, activated muscles secrete myokines and cytokines such as IL-6 and TNFα which, under some circumstances and at low levels, have been shown to have anti-inflammatory effects (Bruunsgaard, 2005).

Unfortunately, there is a lack of similar data in CV patients, e.g., combined effect of n-3 PUFAs and exercise on immune status in CV patients has not been studied yet.

## n-3 Pufas Enhance Endurance Capacity During Exercise in Animal Models and in Humans

Numerous studies show that n-3 PUFAs may have effects on skeletal muscle metabolism (You et al., 2010; Smith et al., 2015), indicating that n-3 PUFAs regulate signaling pathways related to growth and hypertrophy (Smith et al., 2011a), while animal studies demonstrated that n-3 PUFAs diminished skeletal muscle protein breakdown (Whitehouse et al., 2001). Gingras et al. (2007) demonstrated that food enriched in menhaden oil enhances the activation of anabolic signaling proteins in bovine muscle, and muscle protein synthesis is increased as well (Smith et al., 2011a). Consumption n-3 PUFAs may increase some pathways involved in modulation of mitochondrial activity and extracellular matrix organization and decrease the inhibition of mTOR, which is the key anabolic regulator (Yoshino et al., 2016). Animal studies suggest that transcriptional changes in muscle are responsible for anabolic effects of n-3 PUFAs, such as Akt/FOXO, TLR4, and NOD signaling (Castillero et al., 2009; Liu et al., 2013) and enhance the gene expression of key modulators of mitochondrial function (Baillie et al., 1999; Philp et al., 2015).

Results of an vitro study show a general amelioration in energy metabolism of skeletal muscle after n-3 PUFA intake (Hessvik et al., 2010), and a recent animal study on mice has showed that n-3 PUFAs inhibit metabolic dysfunction in skeletal muscle, since a high saturated fat intake increases accumulation of lipids in red muscle, which in turn increases mediators of lipolysis, oxidation, and thermogenesis (e.g., fatty acid transporter genes Fatp4 and Fat/Cd36, fatty acid storage-related gene Dgat1 and Hsl were increased) and suppresses lipogenic genes (Philp et al., 2015).

Furthermore, n-3 PUFAs can modify the membrane fluidity of proteins and lipids within the cell membrane, reducing oxygen consumption and thus improving endurance capacity (Philpott et al., 2019). Different studies on healthy animals (for example birds – semipalmated sandpipers Calidris pusilla or rats) showed that n-3 PUFA supplementation supports recovery of muscle damage caused by exercise and changes muscle lipid composition (Ayre and Hulbert, 1997; Ruf et al., 2006; Maillet and Weber, 2007; Nagahuedi et al., 2009). Similarly, n-3 PUFAs prevent metabolic dysfunction in mice skeletal muscle by limiting the accumulation of intramyocellular lipids in type I muscle fibers (Philp et al., 2015).

It seems that enhanced aerobic capacity is the result of significant exploitation of n-3 PUFAs, which have similar effects in mammalian cells *in vitro* and *in vivo* (Nagahuedi et al., 2009). Certain animals, like migratory birds, salmons and rats, when consuming n-3 PUFAs enriched foods exhibited increased endurance and a large increase in aerobic capacity (“natural doping”) (Ayre and Hulbert, 1997; McKenzie et al., 1998; Guglielmo et al., 2002; Maillet and Weber, 2007). Natural doping is mediated by the incorporation of n-3 PUFAs into the membrane phospholipids and their binding to nuclear receptors which are necessary for regulation of genes which control lipid metabolism. In addition to that, n-3 PUFAs participate in oxygen delivery to skeletal muscle and increase exercise performance (Bruckner et al., 1987). Daily supplementation of n-3 PUFAs in older people, in combination with physical activity, resulted in a significant increase in muscle strength (Rodacki et al., 2012; Smith et al., 2015). Moreover, it has been demonstrated that consumption of 3 g/day of n-3 PUFAs decreases eccentric exercise-induced soreness as a marker of inflammation and minimizes the severe DOMS that results from strenuous strength exercise (Jouris et al., 2011). Supplementation of n-3 PUFAs decreased post-exercise soreness in healthy adults (Jouris et al., 2011) manifested as decreased blood markers of muscle damage and inflammatory markers (Mickleborough et al., 2015).

### Combined Effect of n-3 PUFAs and Exercise on Cardiac Function and Endurance Capacity in Healthy Humans and CV Patients

There is a lack of well-controlled studies on the interaction of n-3 PUFA intake and exercise, particularly in CV patients. In regard to healthy humans, an increase in anaerobic endurance capacity (but not in 20 m sprint performance), explosive leg power, and 1RM knee extensor strength was observed in competitive soccer players who took n-3 PUFA supplementation during 4 weeks of training, suggesting an interaction of n-3 PUFA intake with exercise that requires further study (Gravina et al., 2017). Also, there is a certain level of interaction of dietary n-3 PUFA intake and the level of concomitant habitual exercise in healthy adult subjects, benefiting cardiac autonomic control, measured as heart rate variability (Harbaugh et al., 2013).

Fish oil-enriched diet might cause changes in some systemic hemodynamic parameters and cardiac function (e.g., mean aortic pressure and heart rate) related to stress (moderate exercise), suggesting increased production of endothelium-derived relaxing factor (Lortet and Verger, 1995). For example, rats that were consuming fish oil had decreased vascular resistance (Demaison et al., 2000). Also, n-3 PUFAs exhibited several potential anti-arrhythmic effects in animal models (Den Ruijter et al., 2007). Furthermore, a cross-sectional study in 992 patients with stable coronary artery disease demonstrated that levels of n-3 PUFAs (DHA + EPA) were strongly associated with heart rate recovery, exercise capacity, and exercise time. Increased levels of n-3 PUFAs were also associated with decreased risk of impaired heart rate recovery and exercise time in these patients (Moyers et al., 2011).

## Effects of n-3 Pufas on the Skeletomuscular System in Healthy People

Skeletal muscle is a plastic tissue, sensitive to changes in dietary lipids, capable of adapting to diet and physical activity, with a high level of ability to alter its phenotype, depending on prior nutritional status of the muscle. As the main component of cellular membranes, n-3 PUFAs participate in enzyme regulation and act as signaling molecules (Jeromson et al., 2015).

Increased physical activity, such as intense training, competition situations in elite athletes and rehabilitation physiotherapy (e.g., in CV patients), is followed by muscle microtrauma, oxidative stress, inflammation, neutrophilia, dehydration, lactic acid accumulation, fatigue of central nervous tissue, nutrient stores catabolism, and soreness. Muscle stem cells, tissue satellite cells, are important for the process of growth and recovery of skeletal muscles, reacting to the regeneration process due to mechanical stress induced by exercise (Hawley et al., 2014; Tachtsis et al., 2018). Potential consequences of reducing inflammation after exercise would be faster recovery time, pain reduction, minimization of post-exercise pain, facilitation of exercise training in individuals ranging from patients who are starting exercise programs, medical treatments (physical therapy, cardiac rehabilitation), or athletes undergoing heavy conditioning (Hawley et al., 2014; Tachtsis et al., 2018). Anti-inflammatory effects of n-3 PUFAs could promote muscle stem cell responsiveness to injury by attenuating systemic inflammation (Apolinário et al., 2015; Mackey et al., 2016).

Athletes’ diet may contain n-3 PUFAs considering that they may have an impact on muscle remodeling, muscle recovery, and immune surveillance; however, a small number of studies were conducted in elite athletes. Studies in young and middle-aged (Smith et al., 2011b) or older adults (Smith et al., 2011a) have demonstrated that 8 weeks of n-3 PUFA supplementation (1.86 g of EPA and 1.50 g of DHA) increased muscle protein synthesis rates by sensitizing skeletal muscle to potent anabolic stimuli, such as amino acids and insulin. This effect of n-3 PUFAs includes direct incorporation of n-3 PUFAs into the muscle phospholipid membrane (Smith et al., 2011b; McGlory et al., 2014). Another interesting finding is that n-3 PUFAs may have an effect on preventing and faster healing of slighter soft-tissue injuries caused by exercise (Calder, 2012). Direct incorporation of n-3 PUFAs in the muscle cell membrane (McGlory et al., 2014) and their ability to modify the structural integrity of the cell membrane, together with anti-inflammatory properties of n-3 PUFAs, indicate a protective role of n-3 PUFAs in reducing the effect of eccentric muscle strain on muscle damage (Calder, 2012). Several examples confirm that speculation. Patients with rotator cuff-related shoulder pain taking 1.53 g EPA and 1.04 g DHA for 8 weeks exhibited improvements in disability and pain after 3 months (Sandford et al., 2018). The double-blind study of Lewis and Sandford has shown significant pain reduction after 32 days of n-3 PUFA supplementation and administration of antioxidant pills in recreational athletes with tendinopathies (Lewis and Sandford, 2009). Moderate beneficial effect of n-3 PUFAs (551 mg EPA and 551 mg DHA) on muscle soreness with improved explosive power was observed in elite rugby players (Black et al., 2018). In healthy elderly women, a diet containing n-3 PUFAs improved dynamic explosive strength capacity in resistance training (Edholm et al., 2017). However, not all examples had positive effects. For example, there was no effect of n-3 PUFAs on strength, power and speed improvement, although there was an increment in anaerobic endurance capacity in soccer players (Gravina et al., 2017).

The effect of EPA and DHA on muscle mass in humans is limited. While Smith and colleagues reported increment in muscle protein synthesis after 1.86 g of EPA and 1.5 g of DHA per day for 8 weeks (Smith et al., 2011b), and increment in tight muscle mass in a group of elderly persons (Smith et al., 2015),the same effect was not observed in young athletes. In summary, positive effects of n-3 PUFAs on muscle mass are demonstrated in sedentary people, while effects in trained subjects are still unclear. Improvement in muscle function was also observed by using electromyography and measuring electrical mechanical delay in sedentary and trained people (Rodacki et al., 2012; Lewis et al., 2015). The use of EPA and DHA can have an effect after training on neuromuscular adaptation by implementing n-3 PUFAs in muscle and nerve cells (Bourre, 1989), resulting in improvement of acetylcholine sensitivity and fluidity of the membrane (Lauritzen et al., 2001; Rodacki et al., 2012; Da Boit et al., 2017).

Most of the previous studies investigated the effect of n-3 PUFAs on inflammatory processes and muscle metabolism during exercise, and only a few evaluated their effect on exercise performance (Armstrong et al., 1991; Balvers et al., 2010; Jouris et al., 2011; Mickleborough et al., 2015; Da Boit et al., 2017). For example, for people starting with new exercise programs in order to improve tolerance to new and/or stressful exercises, for those who are performing more intense exercises to reduce pain and stiffness of the muscles, and thus for greater adaptability to training and preparation for competition, supplementation of DHA in diet is justified (Corder et al., 2016).

Data obtained through studies on humans about n-3 PUFA supplementation and its effects in exercise response are still inconclusive, possibly due to the differences in definitions of heterogeneity in study designs; setting, mode, intensity, and duration of exercise; definition of population; different types, dosage, and duration of n-3 PUFA supplementation; the timing of measurements and selections of biomarkers; individual responsiveness and adherence to exercise and dietary protocols, and variation in disease substrates (Mickleborough et al., 2015; Calvo et al., 2017; Da Boit et al., 2017; Kones et al., 2017).

## The Effect of n-3 Pufa Supplementation on Physical Performance in Cardiovascular Patients

A long-term CV effect of exercise training is a decrease in heart rate and heart rate variability due to increased vagal tone (Routledge et al., 2010). Obesity is a known CV risk factor. Since, it was established that dietary intake enriched with fish oil containing n-3 PUFAs may extend life expectancy, many theses about their anti-obesity properties have arisen. Moreover, studies in animal models consistently report that n-3 PUFAs reduce fat mass, particularly in the retroperitoneal and epididymal regions. However, such effects in humans are still under debate. Moreover, despite the thesis on the anti-obesity effect of n-3 PUFAs, human studies on this issue did not demonstrate their positive effects on adiposity and body composition with certainty, but instead they reported that n-3 PUFAs may not aid weight loss (Albracht-Schulte et al., 2018). Moreover, completely contradictory results were brought by a recent study which demonstrated that a higher n-3/n-6 PUFAs ratio in healthy middle-aged women led to adiposity (increased waist circumference) and higher levels of triglycerides, glucose and insulin (Torres-Castillo et al., 2018). Another study showed that consumption of n-3 PUFAs is not associated with total body fat and body fat distribution in the same group of women (Muka et al., 2017). Large meta-analysis from 2018 on the effect of n-3 PUFAs on primary and secondary prevention of CV diseases, which included a total of 7100 both healthy participants and participants with existing illness from 12 trials, reported that increasing n-3 PUFA intake probably causes slight weight gain (Abdelhamid et al., 2018).

Dietary n-3 PUFA intake combined with lifestyle modifications (including increase in regular exercise) leads to improvement of the clinical signs of peripheral vascular disease (claudication) in these patients (*N* = 24 male patients). Also, significant changes in lipid lipoprotein composition, specifically decreased LDL cholesterol, were observed (Nestares et al., 2003). In patients with non-ischemic dilated cardiomyopathy (*N* = 133, randomized to experimental and placebo group), intake of n-3 PUFAs at a dose of 2 g/day for 12 months increased the left ventricular systolic function and cardiac functional capacity, compared to placebo treatment, which might reduce hospitalizations for heart failure (Nodari et al., 2011). However, there is a lack of data on the combined effect of n-3 PUFAS and exercise on physical performance in CV patients. One may only speculate that they may have positive additive effects, particularly in CV patients that develop cachectic conditions.

## Summary and Conclusion

This manuscript aimed to provide a comprehensive review of less studied effects of diet and lifestyle on hemorheology, inflammation, and vascular function in healthy persons and CV patients. Since n-3 PUFA supplements are widely used in the general population and prescribed to CV patients, the focus of this review was on n-3 PUFAs effects complementary or interactive with physical activity. It turned out that the field is inconsistent, due to a wide spectrum of conducted studies claiming opposite results (from little or no effect on CV parameters to beneficial effects on micro and macrovascular function, inflammation and potentially prevention of thrombosis and atherosclerosis. Considering that CV and metabolic diseases have underlying low-grade inflammation at the level of endothelium, it is of particular interest to evaluate all lifestyle factors that can affect these conditions. In this sense, n-3 PUFAs and exercise appear to be good candidates. Major limitations in analysis were the following: heterogeneous study approach, including (a) human and animal studies using transgenic mice or inbred animals subjected to specific challenge; (b) age and health status of the studied population (obese, suffering of CV, autoimmune, inflammatory disorders, focus on primary or secondary prevention of CV events); (c) acute or chronic and moderate or extreme physical activity; (d) dose, composition, and duration of n-3 PUFA supplementation. Dietary intake of n-3 PUFAs is mainly by supplements and very seldom there are studies on functional (enriched) food involved. Nevertheless, results suggest that there is an open space for evaluating the impact of consumption of n-3 PUFAs and exercise on characteristics of blood composition and hemorheology, antithrombic effects, and microvascular function. In the field of functional food in particular, there is a lack of RCT obtained data. Hence, more translational controlled clinical studies with defined experimental protocol are necessary for better understanding of the effects of n-3 PUFA supplementation in health and disease.

## Author Contributions

All authors wrote and revised the manuscript, edited the figures, and approved the submitted version of the manuscript.

## Conflict of Interest Statement

The authors declare that the research was conducted in the absence of any commercial or financial relationships that could be construed as a potential conflict of interest.
